# The frailty index is a predictor of cause-specific mortality independent of familial effects from midlife onwards: a large cohort study

**DOI:** 10.1186/s12916-019-1331-8

**Published:** 2019-05-15

**Authors:** Xia Li, Alexander Ploner, Ida K. Karlsson, Xingrong Liu, Patrik K. E. Magnusson, Nancy L. Pedersen, Sara Hägg, Juulia Jylhävä

**Affiliations:** 1 The Department of Medical Epidemiology and Biostatistics, Karolinska Institutet, Nobels väg 12A, 17165 Stockholm, Sweden; 2 Institute of Gerontology and Aging Research Network – Jönköping (ARN-J), School of Health and Welfare, Jönköping University, Jönköping, Sweden

**Keywords:** Frailty index, Mortality, Familial effect, Time-varying effect, Attributable fraction

## Abstract

**Background:**

Frailty index (FI) is a well-established predictor of all-cause mortality, but less is known for cause-specific mortality and whether familial effects influence the associations. Middle-aged individuals are also understudied for the association between FI and mortality. Furthermore, the population mortality impact of frailty remains understudied.

**Methods:**

We estimated the predictive value of FI for all-cause and cause-specific mortality, taking into account familial factors, and tested whether the associations are time-dependent. We also assessed the proportion of all-cause and cause-specific deaths that are attributable to increased levels of frailty. We analyzed 42,953 participants from the Screening Across the Lifespan Twin Study (aged 41–95 years at baseline) with up to 20 years’ mortality follow-up. The FI was constructed using 44 health-related items. Deaths due to cardiovascular disease (CVD), respiratory-related causes, and cancer were considered in the cause-specific analysis. Generalized survival models were used in the analysis.

**Results:**

Increased FI was associated with higher risks of all-cause, CVD, and respiratory-related mortality, with the corresponding hazard ratios of 1.28 (1.24, 1.32), 1.31 (1.23, 1.40), and 1.23 (1.11, 1.38) associated with a 10% increase in FI in male single responders, and 1.21 (1.18, 1.25), 1.27 (1.15, 1.34), and 1.26 (1.15, 1.39) in female single responders. No significant associations were observed for cancer mortality. No attenuation of the mortality associations in unrelated individuals was observed when adjusting for familial effects in twin pairs. The associations were time-dependent with relatively greater effects observed in younger ages. Before the age of 80, the proportions of deaths attributable to FI levels > 0.21 were 18.4% of all-cause deaths, 25.4% of CVD deaths, and 20.4% of respiratory-related deaths in men and 19.2% of all-cause deaths, 27.8% of CVD deaths, and 28.5% of respiratory-related deaths in women. After the age of 80, the attributable proportions decreased, most notably for all-cause and CVD mortality.

**Conclusions:**

Increased FI predicts higher risks of all-cause, CVD, and respiratory-related mortality independent of familial effects. Increased FI presents a relatively greater risk factor at midlife than in old age. Increased FI has a significant population mortality impact that is greatest through midlife until the age of 80.

**Electronic supplementary material:**

The online version of this article (10.1186/s12916-019-1331-8) contains supplementary material, which is available to authorized users.

## Introduction

Frailty is a major health concern associated with aging [1]. It is a state of multisystem physiological decline and inability to maintain homeostasis, gradually leading to an increased risk of multiple adverse outcomes, such as falls, hospitalizations, and death [1]. Frailty is also predictive of poor prognosis and post-operative complications in older surgical patients [2]. The two principal models to operationalize the frailty concept are the Rockwood frailty index (FI) and the Fried phenotypic model [3]. The FI defines the level of frailty as the ratio of the number of various health deficits present to the number of deficits considered whereas the FP classifies individuals as non-frail, pre-frail, or frail based on poor grip strength; slow walking speed; low physical activity, exhaustion; and unintentional weight loss. Being a continuous scale measure, the FI provides good sensitivity even at the lower end of the frailty continuum, which facilitates studies in younger individuals [4, 5].

The FI is associated with all-cause mortality in different populations, independent of other major risk factors [6]. The FI-mortality relationship is well-established, particularly among older individuals (> 65 years), but less is known for younger adults and for cause-specific mortality—aspects that may have implications for prevention. Both frailty and risk of death can be influenced by familial background, such as genetic and shared environmental factors, which may confound frailty-mortality associations [7]. However, the extent to which the predictive ability of the FI is affected by familial influences has not been studied. Sex-specific characteristics for the frailty-mortality association have also been suggested, but the matter remains inconclusive [8].

Despite the recognition of frailty as a public health concern [9], its direct population mortality impact has received less attention. One way to assess the public health relevance of frailty is to estimate the proportion of deaths that could be delayed if the levels of frailty were decreased in the population. Assessing the fraction of deaths that are attributable to increased frailty, considering both all-cause and cause-specific mortality as outcomes, would provide more precise information about public health relevance.

Consequently, this study aims to estimate the predictive value of the FI on all-cause and cause-specific mortality, separately for men and women. We assess the associations among both unrelated individuals and within twin pairs, to account for familial effects and to assess the degree to which frailty-mortality associations in the general population could be due to shared familial confounding. Secondly, we estimate FI-mortality associations in a time-dependent manner to elucidate the predictive value of FI from midlife and onwards. Lastly, we investigate the number of deaths that could be attributable to increased population levels of frailty by estimating generalized attributable fractions (GAFs) [10–12].

## Methods

### Study population

Data of the present study came from Screening Across the Lifespan Twin Study (SALT), which is part of the Swedish Twin Registry (STR) [13]. In 1998–2002, a total of 44,919 twins born between 1896 and 1958 were interviewed by a structured, computer-assisted telephone interview to collect information about illnesses and health, prescription and nonprescription medication use, occupation, education, and lifestyle behaviors. Twins were also asked to go to their local health care centers and provide blood for analyses of clinical chemistries and zygosity determination.

In the present study, we excluded participants who had more than 20% missing data across the 44 frailty items, and those for whom information on cause-specific mortality could not be retrieved. Eventually, 42,953 twin individuals aged from 41 to 95 years were included in the study, including 11,087 twin individuals whose partner did not respond, 11,548 opposite-sex dizygotic (DZ) twin individuals (5774 twin pairs), 11,812 same-sex DZ twin individuals (5906 twin pairs), and 8506 monozygotic (MZ) twin individuals (4253 twin pairs). Participants were further grouped into single responders, same-sex dizygotic (DZ) twin pairs, and monozygotic (MZ) twin pairs in the analyses, separately for men and women (Additional file 1: Table S1). In the sex-specific analyses, single responders included twins whose partner did not respond in SALT, twins from opposite-sex twin pairs, and one randomly selected member of each same-sex twin pair.

### Assessment of the FI

The FI in SALT was constructed based on self-reported data using the Rockwood deficit accumulation model according to a standard procedure [4]. Briefly, the deficits to be included in the FI had to have a ≥ 1% prevalence and ≤ 10% missingness rate in the study population. Forty-four symptoms, signs, disabilities, and diseases covering a wide range of biological systems and associated with health status were considered in the FI. The items and scoring of the deficits are presented in Additional file 1: Table S2. Imputation was used to replace missing values in order to maximize the utilization of the data. Specific methods for the imputation and sensitivity analysis for the imputed data are presented in Additional file 1: Appendix S1.

An FI value for each individual was calculated as the number of deficits present divided by the total number of deficits (*n* = 44). For example, an individual having 8 deficits has an FI of 8/44 = 0.18. To assess the validity of FI in SALT, we examined the distribution and associations with age. The FI was treated as a continuous variable with 10% increase per unit when modeling survival (hazard ratio, HR).

### Assessment of mortality

All-cause mortality data, including dates of deaths, were obtained from linkages of the STR to Swedish national registers through the personal identification number assigned to all residents. All-cause mortality data used in this study were updated on December 31, 2017, yielding up to 20 years of follow-up.

Cause-specific mortality data were obtained from the Cause of Death Register (CDR), with the latest update on December 31, 2014, yielding up to 17 years of follow-up. The CDR records include information about the underlying and contributory causes of death for all individuals registered as Swedish residents in the year of their death. Causes of death were recorded using the International Classification of Diseases (ICD) codes, with ICD-10 from 1997 and onwards. We considered cardiovascular disease (CVD; including stroke), respiratory-related causes, and cancer as the specific causes of death. The ICD codes included in each cause and the consensus classification used when more than one cause of death was recorded are presented in Additional file 1: Tables S3 and S4, respectively.

### Assessment of covariates

Years of education, tobacco use status, body mass index (BMI), and history of diseases were assessed from the self-reported data in SALT. Tobacco status was categorized as non-user (reference category) or user if the person was currently smoking or using smokeless tobacco regularly or had previously smoked or used smokeless tobacco regularly. BMI was calculated as weight divided by height squared (kg/m^2^). Individuals were classified as having a history of CVD, chronic respiratory diseases, and/or cancer, if he/she reported any of the following conditions respectively: (1) angina pectoris, myocardial infarction, heart failure, stroke, high blood pressure, claudication, irregular cardiac rhythm/atrial fibrillation, circulation problems in limbs, or thrombosis, (2) asthma or chronic lung disease, (3) cancer, tumor disease, or leukemia.

### Statistical methods

We used generalized survival models (GSMs) for the association between FI and mortality [14, 15]. Briefly, these are a generalization of flexible parametric survival models where the underlying baseline hazard is fitted as a smooth spline term, in our case via a natural cubic spline with 3 to 5 degrees of freedom for different outcomes and cohorts. We chose GSMs over conventional Cox regression for both computational and conceptual reasons—faster model fit for time-varying effects, easier estimation and extraction of time-varying effects and survival function from the fitted model, but also less restrictive assumptions when estimating attributable fractions/generalized impact fractions than for the Cox model [16]. Given the wide age range and long follow-up among participants, we used attained age as underlying time scale for all models to eliminate the possibility of non-proportional effects of FI that were driven by chronological age. Subjects in the cause-specific analyses were censored at the date when they died from other causes than the current outcome of interest, or at end of follow-up.

We first tested the association with survival assuming a time-constant effect among single responders, same-sex DZ twin pairs, and MZ twin pairs (see Additional file 1: Table S1 for grouping). Next, we tested if there were significant time-dependent effects for those causes of death that were significant in the time-constant model among single responders. To elucidate sex-specific characteristics of the associations, all analyses were performed separately for men and women. Years of education, tobacco use, and BMI were included as covariates in all models. For the cause-specific analyses, history of cancer, CVD, or chronic respiratory diseases at baseline were included as a covariate when the corresponding cause of death was the outcome of interest, see Additional file 1: Appendix S2, Eq.1, for the basic models for unrelated individuals.

For the same-sex DZ and MZ twin pairs, a between-within decomposition along with a random effect from a gamma distribution was introduced to the GSM, which allow us to adjust for shared familial effects both due to shared patterns of exposure and confounders included in the model as well as general unmeasured similarity in survival patterns in twin pairs [16, 17]. We have previously used a similar model in twin data for telomere length and survival [18]. The model is described in detail in Additional file 1: Appendix S2, Eq. 2.

For the subgroup of single responders, we also included interaction terms that allow the association between FI and survival to vary with age at assessment as well as time since assessment; details are presented in Additional file 1: Appendix S2, Eq. 3.

The generalized attributable fraction (GAF), in the literature also referred to as generalized impact fraction [12], was used to quantify the potential public health impact of FI on the survival outcome based on the time-constant survival models [11, 12, 16]. The GAF is an integrated predictive measure that takes into account both the distribution of a risk factor in the population as well as the strength of association between risk factor and outcome. Unlike the classical attributable fraction, it is also defined for a continuous exposure, eliminating the need to re-fit our models with dichotomized FI as exposure. In this study, we report the GAF function [12, 19], which shows the proportion of deaths that could potentially be prevented before a given age, or in other words, delayed beyond a certain age through a hypothetical intervention. In our approach, we assessed the mortality impact of a hypothetical intervention among those individuals who have an FI above a threshold of (a) 0.10 (i.e., the threshold for “least fit” [20]) and (b) 0.21 (i.e., the threshold for “frail” [20]). That is, the individuals with an FI above these thresholds would be (hypothetically) intervened to reduce their counterfactual FI values just below these thresholds (0.099 or 0.209, respectively) to achieve the given reduction in mortality.

As our definition of FI includes items related to CVD, respiratory diseases, and cancer, we created three “reduced” FIs that were stripped of either CVD-related items (angina pectoris, myocardial infarction, heart failure, stroke, high blood pressure, claudication, irregular cardiac rhythm/atrial fibrillation, circulation problems in limbs, and thrombosis), respiratory-related items (chronic lung diseases and asthma), or cancer. These reduced FIs were then used as exposures in a sensitivity analysis for the corresponding cause-specific survival models. An additional sensitivity analysis using the original 44-item FI was performed for respiratory-related mortality by including only the 17,905 non-tobacco users in the analysis.

*P* values reported are two-sided, and the statistical significance level was set at *p* < 0.05. All analyses were conducted using Stata 15.1 and R 3.4.3. Statistical analyses involving GSMs were implemented using the rstpm2 package 1.4.0 [21].

## Results

Characteristics of the study population are presented in Table 1. Of 42,953 individuals, 46.4% were men and 47.3% were complete twin pairs. At baseline, the median level of FI was 0.097 in men and 0.119 in women. FI distributions were skewed with a long right tail in all groups (total cohort, males, and females) (Additional file 1: Figure S1). The single responders tended to be older, had a lower education, and used tobacco products more frequently (in men only) than those from complete pairs.

**Table 1:** Characteristics of the study population

	Men (*N* = 19,924)			Women (*N* = 23,029)		
	Single responders^a^	DZ twins^b^	MZ twins	Single responders^a^	DZ twins^b^	MZ twins
Number of participants^c^	15,473	5266	3636	17,321	6546	4870
Age at baseline, mean (SD)	58.7 (10.6)	57.4 (9.6)	56.9 (9.2)	59.6 (11.2)	58.5 (10.3)	57.8 (10.2)
BMI (kg/m^2^), mean (SD)	25.5 (3.1)	25.6 (3.1)	25.5 (3)	24.6 (3.7)	24.5 (3.7)	24.4 (3.8)
Education (year), mean (SD)	10.4 (3.2)	10.6 (3.2)	10.8 (3.2)	10.4 (3.2)	10.5 (3.2)	10.8 (3.2)
Tobacco products use^d^, %	64.3	63.1	59.5	53.9	54.3	52.5
FI, median (IQR)	0.097 (0.102)	0.094 (0.097)	0.091 (0.097)	0.125 (0.125)	0.119 (0.125)	0.119 (0.119)
History of diseases at baseline, %						
CVD	35.5	34.0	31.5	37.4	35.6	34.8
Respiratory disease	9.6	8.2	9.6	11.9	10.8	11.7
Cancer	4.7	4.2	4.7	9.1	8.5	9.3
Time to follow-up, median (IQR)						
All-cause mortality	16.7 (2.8)	16.9 (2.4)	16.9 (2.3)	16.9 (2.6)	17.0 (2.5)	17.1 (2.4)
Cause-specific mortality	14.0 (2.5)	14.1 (2.3)	14.1 (2.3)	14.2 (2.5)	14.3 (2.2)	14.3 (2.3)
Death during the follow-up, %						
All causes	31.3	26.0	23.4	28.3	25.0	22.1
CVD	8.9	7.0	5.7	7.4	6.1	4.8
Respiratory-related causes	2.7	2.1	1.9	2.4	2.2	1.7
Cancer	9.0	7.7	7.5	7.0	6.7	5.9

^a^Single responders included twins whose partner did not respond in SALT, twins from opposite-sex twin pairs, and one randomly selected member of each same-sex twin pair

^b^DZ twins refer to the same-sex DZ twins

^c^Number of participants refers to the number of single responders or the number of individuals in DZ/MZ twin pairs

^d^Participants who used tobacco products include current smokers, ex-smokers, and snuff users at baseline

**Fig. 1:**
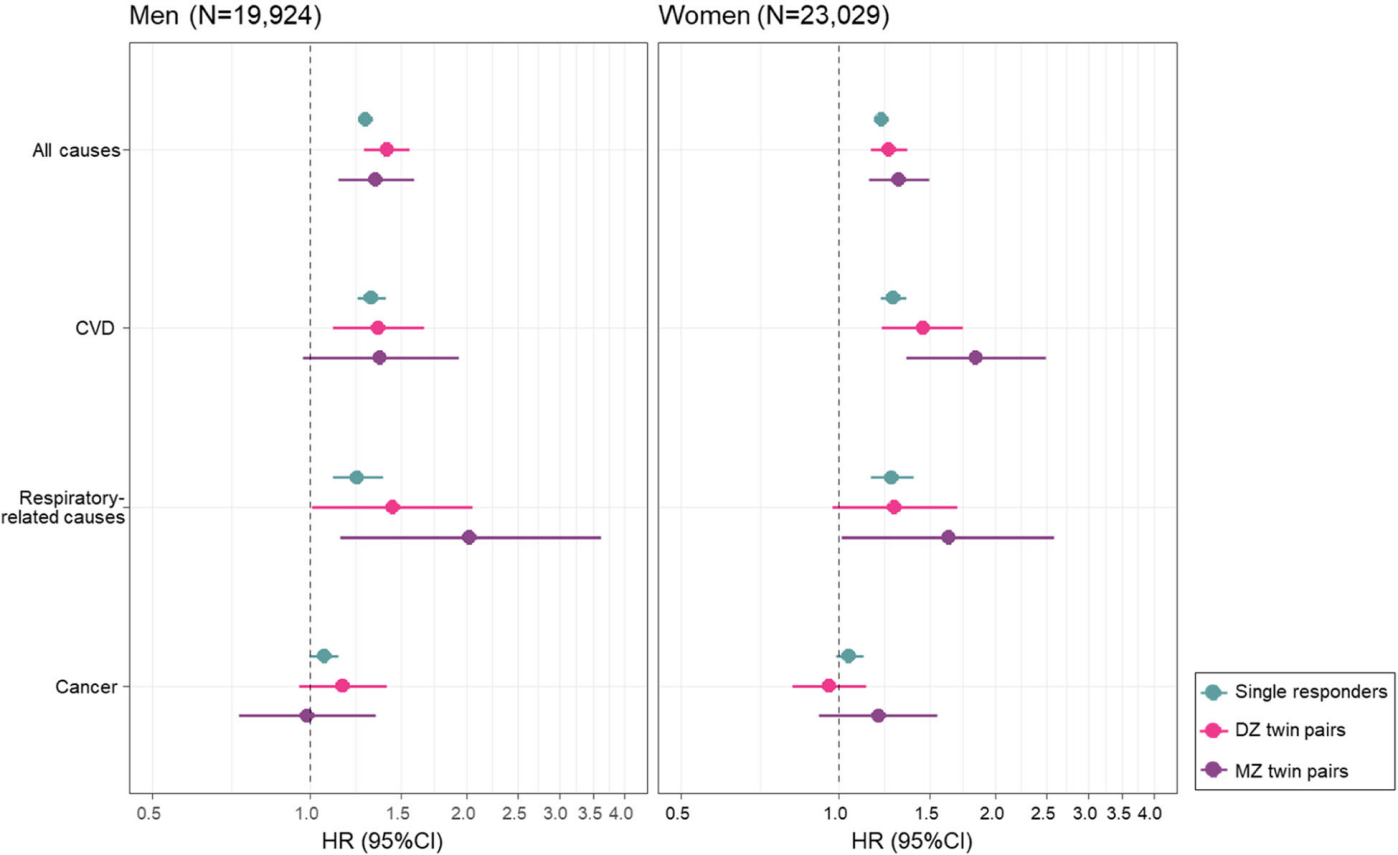
Time-constant associations between a 10% increase in the FI and all-cause and cause-specific mortality. Increased FI significantly predicted higher risks of deaths due to all causes, CVD, and respiratory-related causes in time-constant models in both men and women. No significant associations were observed for cancer mortality. The associations of FI did not exhibit marked differences among single responders, same-sex DZ twins, and MZ twins, and the associations were also similar in men and women. All models considered attained age as time scale, adjusted for BMI, years of education, and tobacco use status, and additionally adjusted for history of CVD, respiratory diseases, or cancer in corresponding cause-specific mortality analysis

**Fig. 2:**
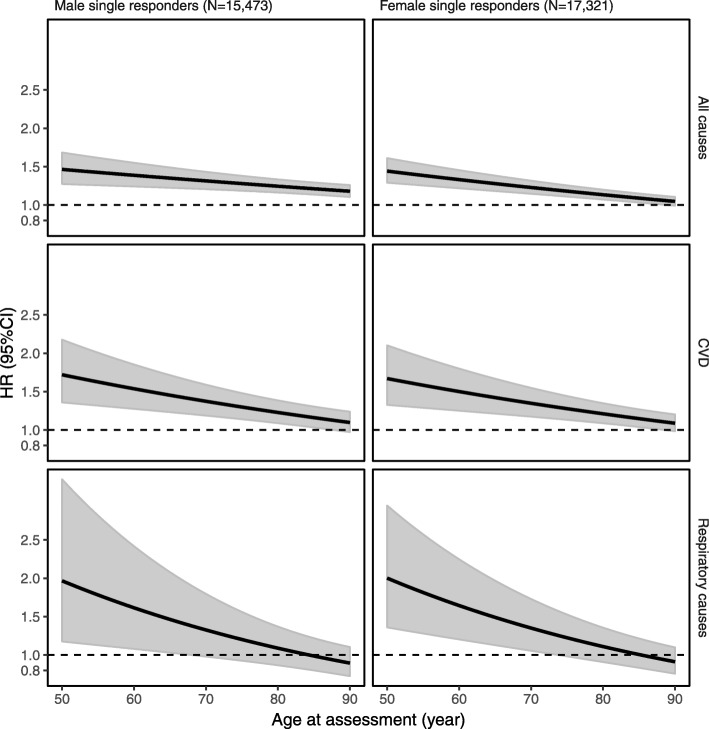
Time-dependent associations between a 10% increase in the FI and mortality in single responders. Time-dependent effects of FI for all-cause, CVD, and respiratory-related mortality in both men and women were observed, with relatively greater HRs at younger ages and effect sizes decreasing with increasing age at FI assessment for all the causes. All models were adjusted for BMI, years of education, and tobacco use status, and additionally adjusted for history of CVD or respiratory diseases in corresponding cause-specific mortality analysis, and were fitted as a function of age at FI assessment

During 20 years of follow-up for all-cause mortality and 17 years of follow-up for cause-specific mortality since 1998–2002, 12,222 (28.5%) deaths were recorded, with 3270 (7.6%), 1051 (2.4%), and 3302 (7.7%) deaths due to CVD, respiratory-related causes, and cancer, respectively. After controlling for education, tobacco use, BMI, the history of corresponding diseases, and familial effects in twins, increased FI significantly predicted higher risks of deaths due to all causes, CVD, and respiratory-related causes in time-constant models in both men and women (Fig. 1). The between effects from these models for twin pairs showed no significant associations, except for CVD mortality in MZ women (Additional file 1: Table S5). The corresponding hazard ratios (HRs) associated with a 10% increase in the FI were 1.28 (1.24, 1.32), 1.31 (1.23, 1.40), and 1.23 (1.11, 1.38) in male single responders and 1.21 (1.18, 1.25), 1.27 (1.15, 1.34), and 1.26 (1.15, 1.39) in female single responders (Additional file 1: Table S5). No significant associations were observed for cancer mortality (Fig. 1). The associations between FI and mortality did not exhibit marked differences among single responders, same-sex DZ twins, and MZ twins and were also similar in men and women (Fig. 1). Additionally, we found time-dependent effects of FI for all-cause, CVD, and respiratory-related mortality in both men and women, with relatively greater HRs at younger ages and effect sizes decreasing with increasing age at FI assessment for all the causes (Fig. 2, single responders only), with a 1–2% decrease in strength of the association per extra year of age at assessment (Additional file 1: Table S6; single responders only).

Sensitivity analyses demonstrated that the association of FI and cause-specific mortality did not change appreciably when FI was stripped of item(s) related to the cause of death under examination, or when tobacco users were excluded from the study population (Additional file 1: Table S7).

**Fig. 3:**
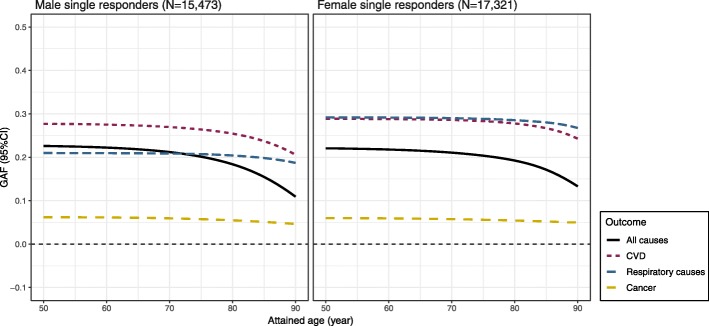
Generalized attributable fraction (GAF) functions for all-cause and cause-specific mortality in single responders. The GAF represents the proportion of deaths that could be prevented before a given age (i.e., delayed beyond that age) if FI levels greater than 0.21 were reduced to the level of 0.209 in the population. The estimated GAFs demonstrated that theoretical reduction of the FI levels below 0.20 would potentially delay 20–30% of all-cause, CVD, and respiratory-related deaths in men and women beyond the age of 80 years. All models were adjusted for BMI, years of education, and tobacco use status, and additionally adjusted for history of CVD, or respiratory diseases in corresponding cause-specific mortality analysis, and were fitted as a function of age at FI assessment

The GAF estimates across the whole age range are presented in Fig. 3, and the GAFs (95%CIs) before the age of 80 years are also provided in a numeric format in Additional file 1: Table S8. The GAFs demonstrated a decreasing trend towards the oldest ages and were relatively constant before the age of 80. In men, the GAFs (95%CI) revealed that 18.4% (16.1–20.7%) of all-cause deaths, 25.4% (20.1–30.8%) of CVD deaths, and 20.4% (10.4–30.5%) of respiratory-related deaths could be delayed beyond age 80 if the FI levels > 0.21 were reduced in the population. In women, the corresponding GAFs were 19.2% (16.6–21.9%) for all-cause deaths, 27.8% (22–33.5%) for CVD deaths, and 28.5% (18.4–38.7%) for respiratory-related deaths (Additional file 1: Table S8).

## Discussion

In this study, we constructed the FI based on the Rockwood cumulative deficit model in a Swedish twin population and assessed its predictive value on all-cause and cause-specific mortality. During the 20-year follow-up, an increase in FI conferred a significant risk for all-cause, CVD, and respiratory-related mortality, in both men and women. Relatively greater HRs were observed at younger ages; the relative risk conferred by a 10% increase in the FI was greatest at the attained age of 50 after which it decreased by 1–2% per year. The associations were persistent when adjusted for tobacco use, education, and BMI. When adjusting for familial effects in twin pairs, we found no attenuation of the associations between FI and mortality in comparison to those observed in single responders. In fact, even an increasing trend in the strength for FI and respiratory-related mortality in men and for FI and CVD mortality in women was found when the familial effects were accounted for. No associations with cancer mortality were however found. The estimated GAFs demonstrated that a (theoretical) reduction of the FI levels below 0.21 could prevent 18.4–28.5% of all-cause, CVD, and respiratory-related deaths in men and women before the age of 80 years. In line with the relatively greater mortality risks observed in younger ages, the GAFs were likewise greater at and across midlife and younger old ages, and decreased towards the oldest ages.

As frailty is considered to be a manifestation of aging, FI in relation to mortality has been mostly investigated in older populations [6, 22]. Several studies with wide age spectra have nevertheless suggested that a negative effect of increased FI on mortality could also be observed in younger adults [20, 23–25]. In fact, previous findings by others [26] and us [23, 25] have suggested that the frailty-mortality association is relatively stronger among the young than the old. In the present study, we modeled the effect in a time-dependent manner and found that for all causes, higher FI was associated with a greater relative risk of mortality at younger ages and the associations attenuated towards the oldest ages. Such accumulating evidence suggests that a shift towards more research into frailty in younger populations is warranted and younger individuals should also be considered for frailty screening.

Although a wealth of evidence exists to demonstrate that a higher level of frailty is predictive of all-cause mortality, previous studies have mostly been conducted in unrelated individuals [6, 21]. The associations may thus be confounded by familial factors, such as genetics and early life environment, affecting the risk of both frailty and mortality. We tested this by modeling the effects in MZ and DZ twin pairs, and found no evidence that the associations were attenuated compared to the results obtained in single responders, and the between models in twins suggested overall very little to no contribution of familiar factors to the associations. In other words, the FI is at least as useful a predictor of mortality after adjustment for shared family history in twins as in a general, unrelated population, suggesting that FI captures the individual association between frailty at time of assessment and risk of mortality with little or no confounding from shared familial lifestyle or genetic factors.

Frailty presents a sex-specific pattern that is similar to the male-female health-survival paradox in which women experience higher rates of disability and comorbidity yet still outlive men [27]. Women have higher levels of frailty throughout the age range, but men are more vulnerable to death at any given level of frailty [4, 8]. However, whether frailty is a stronger risk factor for men than for women is currently unclear and different studies have reported contrasting results [28–32]. We tested this hypothesis by assessing the associations with both all-cause and cause-specific mortality in men and women separately. Overall, the HRs were similar in men and women, even when adjusted for familial effects in twins. The time-dependent effects also demonstrated similar patterns in men and women, with relatively greater HRs observed at midlife and younger old ages and an attenuating trend towards the oldest ages.

Other studies that used the Fried phenotypic model instead of FI to measure frailty have also demonstrated that increased frailty is predictive of CVD mortality: Crow et al. reported an association that was adjusted for sex in an analysis including men and women [33], and Veronese et al. analyzed men and women separately and demonstrated that the association was stronger in women [34]. However, as analyses into cause-specific mortality and frailty are currently limited, more research is warranted to establish the relationship between frailty and cause-specific mortality. Regarding cancer mortality, a study in older breast cancer patients has shown that frailty is predictive of both all-cause and breast cancer mortality [35], and a systematic review has concluded that frailty is an independent predictor of all-cause mortality in older patients with various cancers [36]. Nevertheless, in the light of our finding that no significant association was observed between the FI and cancer mortality, it appears that the level of frailty is not predictive of cancer deaths in the general population of middle-aged and older individuals, most of whom do not have (or have had) cancer at the time of the FI assessment. Our previous results in a smaller STR sample have also shown that the suggestive association found between the FI and cancer mortality is attenuated after adjusting for present cancer diagnosis [23], indicating that the level of frailty might be a valid predictor of cancer mortality only among those who already have cancer.

Due to its association with various adverse outcomes, frailty presents a public health concern. The risk is not restricted to the highest end of the frailty continuum; lower levels of frailty, such as the pre-frailty state measured using the FP, are also predictive of mortality [3]. Therefore, we assessed the proportion of deaths that could be avoided by decreasing the levels of frailty using the cut-offs for “least fit” and “frail” according to Rockwood et al. [20]. We found that before the age of 80, these proportions ranged from approximately 20 to 30% for all the causes, in both men and women, and the GAFs for the threshold of “least fit” were on average only 2% greater than those for “frail”. Hence, the GAFs not only highlight a great potential for preventions aimed at reducing mortality by reducing the levels of FI, but also that most of the benefit from such an intervention would come from reducing the FI levels greater than 0.21. Our GAF estimates for all-cause mortality are higher than previously reported in a systematic review that estimated the population risk of all-cause mortality attributable to frailty to be 3–5%. The systematic review used a higher cut-off, corresponding to the frailty state, and the individuals included were older than 65 years [22]. These differences may account at least in part for the differing results. In fact, the GAFs in our study showed a decreasing trend towards the very old ages, indicating that higher frailty bears a relatively greater population risk of mortality at midlife and younger old ages. A recommendation to screen all individuals at 70 years and older for frailty at health care facilities has been put forward [37]. However, pertaining to mortality risk stratification, our results would suggest a potential benefit of considering also middle-aged individuals for frailty screening.

The present study has several strengths. First, we had a large population of twins that allowed us to control for unmeasured familial effects. Second, contrary to previous research mainly focusing on FI among older individuals (> 65 years), our cohort also included younger adults and had an age range from 40 to 95 years, which allowed us to model the association between frailty and mortality as a continuous function of age at assessment. Third, we had a very long follow-up, up to 20 years for all-cause mortality and up to 17 years for cause-specific mortality, which enables us to draw conclusions on the long-term predictive ability of the FI. Indeed, the associations in this study demonstrate that the FI is predictive of mortality in a long-term follow-up and align with a previous study by our group, in which we demonstrated that the FI is predictive of all-cause mortality for up to 30 years [23].

The present study also has some limitations. Frailty was measured using only one scale, the FI, and was based on self-reported data. Another limitation is that many of the included FI items were diseases or symptoms, making medical conditions having a relatively large weight in our FI. As our FI contained items of cancer, CVD, and chronic respiratory diseases, in the cause-specific analysis, we adjusted each of the models for the history of the given disease that was the cause of death in that analysis. For each of the cause-specific mortality analyses, we additionally created a modified FI that was stripped of the item(s) related to the cause of death under examination. Lastly, we repeated the analysis of respiratory-related mortality in non-tobacco users. All results remained unchanged in these sensitivity analyses, suggesting that the FI is a robust marker for predicting the risk of death due to CVD and respiratory-related causes, even in the presence of these diseases.

## Conclusions

An increase in FI predicted higher risks of all-cause, CVD, and respiratory-related mortality, independent of familial effects, and exhibited time-dependent associations suggesting that a higher level of frailty is a relatively greater risk factor for middle-aged than for older individuals. Our results also highlight the significant population mortality impact of increased FI levels, especially above the threshold commonly considered to demarcate a “frail” state.

## Additional file

Additional file 1: **Table S1.** Grouping of the study population. **Table S2.** The 44 frailty items and the coding rules. **Table S3.** ICD codes used to classify the cause-specific mortality. **Table S4.** Consensus classification for the cause-specific mortality when more than one cause of death was recorded. **Table S5.** Time-constant associations between 10% increase in the FI and all-cause and cause-specific mortality. **Table S6.** Assessment of time-dependent associations between FI and mortality in single responders. **Table S7.** Sensitivity analyses on the associations between 10% FI increase on all-cause and cause-specific mortality in single responders. **Table S8.** Generalized attributable fractions (%, 95%CI) for all-cause and cause-specific mortality in single responders at the attained age of 80. **Table S9.** Generalized attributable fractions (%, 95%CI) for all-cause and cause-specific mortality in twins at the attained age of 80. **Figure S1.** Distribution of the frailty index in our sample. Appendix S1 Imputation of missing values. Appendix S2 Specification of generalized survival models (GSM) fitted to the data. (DOCX 66 kb)

## Abbreviations

BMI: Body mass index
CDR: Cause of death register
CVD: Cardiovascular disease
DZ twins: Dizygotic twins
FI: Frailty index
GAF: Generalized attributable fractions
GSM: Generalized survival model
HR (95%CI): Hazard ratio (95% confidence interval)
HR: Hazard ratio
ICD: International Classification of Diseases
MZ twins: Monozygotic twins
SALT: Screening Across the Lifespan Twin Study
STR: Swedish Twin Registry
